# The APE1 Asp/Asp Genotype and the Combination of APE1 Asp/Asp and hOGG1-Cys Variants Are Associated With Increased p53 Mutation in Non–Small Cell Lung Cancer

**DOI:** 10.2188/jea.JE20120048

**Published:** 2012-11-05

**Authors:** Chun-Hsuan Lin, Po-Ming Chen, Ya-Wen Cheng, Chih-Yi Chen, Chiun-Jye Yuan, Huei Lee

**Affiliations:** 1 Department of Biological Science and Technology, National Chiao Tung University, Hsinchu, Taiwan, ROC; 2 Institute of Medicine, Chung Shan Medical University, Taichung, Taiwan, ROC; 3 Graduate Institute of Cancer Biology and Drug Discovery, Taipei Medical University, Taipei, Taiwan, ROC; 4 Department of Surgery, China Medical University Hospital, Taichung, Taiwan, ROC

**Keywords:** hOGG1 Ser326Cys, APE1 Asp148Glu, p53 mutation, NSCLC

## Abstract

**Background:**

The hOGG1 Ser326Cys polymorphism is associated with lung cancer risk, but there are limited data regarding an association between the APE1 Asp148Glu polymorphism and lung cancer. Biological evidence shows that the hOGG1-Cys allele results in less DNA repair activity; however, this is not associated with p53 mutation in lung cancer. Therefore, we investigated whether an interaction between hOGG1 and APE1 is associated with the frequency of p53 mutation in lung cancer.

**Methods:**

We studied 217 Taiwanese adults with primary lung cancer. DNA polymorphisms of hOGG1 and APE1 were determined by polymerase chain reaction (PCR)-based restriction fragment length polymorphism. Mutations in p53 exons 5–8 were detected by direct sequencing. Multiple logistic regression was used to estimate odds ratios (ORs) and 95% CIs for the risk of p53 mutation associated with polymorphisms of hOGG1 and APE1 in lung cancer.

**Results:**

As expected, no association between hOGG1 polymorphism and p53 mutation was observed in this population. However, a higher risk of p53 mutation was found in participants with the APE1 Asp/Asp genotype than in those with the APE1-Glu allele (OR, 2.15; 95% CI, 1.19–3.87; *P* = 0.011). The risk of p53 mutation was also higher in participants with APE1 Asp/Asp plus hOGG1-Cys than in those with APE1-Glu plus hOGG1 Ser/Ser (OR, 3.72; 95% CI, 1.33–10.40; *P* = 0.012).

**Conclusions:**

These results suggest that the APE1 Asp/Asp genotype and the combination of the APE1 Asp/Asp and hOGG1-Cys variants are associated with increased risk of p53 mutation in non–small cell lung cancer.

## INTRODUCTION

In Taiwan, lung cancer is the leading cause of cancer death among women and the second leading cause among men.^1^ Cigarette smoking is considered to be the primary cause of lung cancer; however, less than 20% of smokers develop lung cancer.^2^^,^^3^ Thus, it is possible that different individuals have different capacities for detoxification and DNA repair. Exposure to tobacco smoke might induce oxidative DNA damage that, when combined with impaired DNA repair, may increase lung cancer risk.^4^^–^^7^

Reactive oxygen species (ROS) are linked to chronic inflammation and lung cancer development.^8^^,^^9^ Oxidative reactions due to ROS give rise to DNA modifications, which are then primarily repaired via a DNA base excision repair pathway. DNA repair is initiated by excision of damaged bases by specific DNA glycosylases, such as human 8-oxoguanine-DNA glycosylase (hOGG1) and apurinic/apyrimidinic endonuclease 1 (APE1).^10^^–^^12^ Research has shown that the product of the Ser326 allele of hOGG1 has an increased capacity for removing 8-hydroxyguanin and a high binding affinity for DNA.^13^^,^^14^ In contrast, a biochemical assay showed that the product of the APE1 Asp148Glu allele had similar endonuclease and DNA binding activity to the wild-type allele.^15^

The association of the base excision repair genes hOGG1 and APE1 with lung cancer risk had been extensively investigated. Most of these studies were case-control studies that found an association between hOGG1 Ser326Cys polymorphism and increased lung cancer risk^16^^–^^20^; however, most found no association between APE1 Asp148Glu polymorphism and lung cancer risk.^16^^,^^21^^–^^23^ The exception was 1 report that showed that APE1 Asp148Glu polymorphism was associated with lung cancer risk after adjusting for age, sex, and smoking.^24^ Interestingly, the hOGG1 and APE1 genetic polymorphisms were significantly associated with lung cancer risk in heavy smokers but not in light smokers or nonsmokers.^16^^–^^22^

p53 mutation, which is induced by carcinogens in cigarette smoke, frequently occurs in exons 5–8. This is the principal known carcinogenic pathway involved in lung cancer among smokers.^25^^,^^26^ At this time, there is no molecular evidence to indicate any similar association of hOGG1 Ser326Cys or APE1Asp148Glu with lung cancer risk.

In the present study, we obtained tissue samples from lung tumors and adjacent normal lung from 217 Taiwanese patients with lung cancer. We examined p53 mutation in lung tumors by direct sequencing and used polymerase chain reaction-restriction fragment length polymorphism (PCR-RFLP) to determine the existing genetic polymorphisms of hOGG1 and APE1 in the normal lung tissue. Genotyping analysis of both genes was confirmed in control subjects (*n* = 206) and accorded with the Hardy–Weinberg equilibrium (eTable 1). The aim of this study was to determine whether the hOGG1 Ser326Cys and APE1Asp148Glu genetic polymorphisms were associated with increased p53 mutation in lung cancer.

## METHODS

### Participants

From 1993 to 2005, surgical specimens were obtained from 217 patients at Taichung Veterans General Hospital, Taichung, Taiwan, ROC who had histologically or cytologically confirmed primary non–small cell lung cancer (NSCLC). All participants were older than 18 years and had incident primary lung cancer. Age ranged from 38 to 84 years. Written informed consent for the use of tumor specimens was obtained from each participant before surgery, as required by the Institutional Review Board at Taichung Veteran’s General Hospital. Demographic data for each individual, including age, sex, tumor type and stage, and smoking status, were collected by interview and a review of hospital charts. Smokers were defined as current or previous smokers, and nonsmokers were defined as individuals who had never smoked.

### Genotyping of the hOGG1 and APE1 polymorphisms

Genotyping analysis of the hOGG1 Ser326Cys (C1245G) and APE1 Asp148Glu (T1350G) polymorphism from genomic DNA samples of adjacent normal lung tissue was conducted by PCR-RFLP as described previously.^17^^,^^21^

### Direct sequencing of the p53 gene

To identify mutations in exons 5–8 of the p53 gene, an autosequencer (ABI 3100-Avant Genetic Analyzer; Applied Biosystems, Foster City, CA, USA) was used for direct sequencing of PCR products amplified from lung tumor tissues as described previously.^27^^,^^28^

### Statistical analysis

Statistical analysis was performed using SPSS statistical software (Version 13.0 SPSS Inc., Chicago, IL, USA). Multiple logistic regression was also used to estimate odds ratio (ORs) and 95% CIs for the risk of increased mutation p53, as compared with the wild type, in lung cancer. A *P* value less than 0.05 was considered statistically significant.

## RESULTS

### Association of hOGG1 Ser326Cys and APE1 Asp148Glu with p53 mutation in lung cancer

PCR-RFLP analysis was used to examine the hOGG1 Ser326Cys and APE1 Asp148Glu polymorphisms. The association of the hOGG1 and APE1 polymorphisms with clinicopathologic parameters in Taiwanese with lung cancer is shown in Table 1. No association with clinical parameters was seen for either gene polymorphism, except that men had a somewhat higher frequency than women of the APE1 Asp/Asp genotype (*P* = 0.049). Direct sequencing analysis showed that 97 of 217 (44.7%) lung tumor samples had p53 mutations. The frequencies of p53 mutation in men, smokers, and in squamous cell carcinomas were significantly higher than in women (*P* = 0.003), nonsmokers (*P* = 0.004), and adenocarcinomas (*P* = 0.001; Table 2), respectively. In addition, the frequency of p53 mutation was higher in participants with late-stage tumors (II and III) than in those with early-stage tumors (I) (57% for stage II, 49% for stage III vs 34% for stage I).

**Table 1.** Associations between clinical parameters and genotypes

Characteristics	No.	hOGG1		*P*	APE1		*P*
	
		Ser/Ser	Ser/Cys + Cys/Cys		Asp/Asp	Asp/Glu + Glu/Glu	
		No. (%)	No. (%)		No. (%)	No. (%)	
Age, y							
<66	108	28 (26)	80 (74)	0.608	42 (39)	66 (61)	0.298
≥66	109	25 (23)	84 (77)		50 (46)	59 (54)	
Sex							
Female	65	14 (22)	51 (78)	0.518	21 (32)	44 (68)	0.049
Male	152	39 (26)	113 (74)		71 (47)	81 (53)	
Smoking status							
Nonsmoker	104	26 (25)	78 (75)	0.850	40 (38)	64 (62)	0.260
Smoker	113	27 (24)	86 (76)		52 (46)	61 (54)	
Tumor histology							
Adenocarcinoma	111	28 (25)	83 (75)	0.779	48 (43)	63 (57)	0.796
Squamous carcinoma	106	25 (24)	81 (76)		44 (42)	62 (58)	
Stage							
I	80	18 (23)	62 (77)	0.358	37 (46)	43 (54)	0.624
II	35	6 (17)	29 (83)		13 (37)	22 (63)	
III	102	29 (28)	73 (72)		42 (41)	60 (59)	

*P* values obtained from the chi-square test.

**Table 2.** Association of p53 mutation with clinical characteristics of patients with lung cancer

Characteristics	No.	p53 mutation		OR (95% CI)	*P*
		No	Yes		
		No. (%)	No. (%)		
Age, y					
<66	108	60 (56)	48 (44)	1	0.940
≥66	109	60 (55)	49 (45)	1.02 (0.59–1.74)	
Sex					
Female	65	46 (71)	19 (29)	1	0.003
Male	152	74 (49)	78 (51)	2.55 (1.37–4.15)	
Smoking status					
Nonsmoker	104	68 (65)	36 (35)	1	0.004
Smoker	113	52 (46)	61 (54)	2.22 (1.28–3.83)	
Tumor histology					
Adenocarcinoma	111	74 (67)	37 (33)	1	0.001
Squamous cell carcinoma	106	46 (43)	60 (57)	2.61 (1.50–4.53)	
Stage					
I	80	53 (66)	27 (34)	1	
II	35	15 (42)	20 (57)	2.62 (1.16–5.91)	0.021
III	102	52 (51)	50 (49)	1.89 (1.03–3.46)	0.039

We therefore examined whether p53 mutation was more frequent in participants with high-risk genotypes of hOGG1 and APE1 than in those with low-risk genotypes. p53 mutation in lung tumors of this study population was not associated with hOGG1 polymorphisms. However, participants with the APE1 Asp/Asp genotype had an OR of 2.15 (95% CI, 1.19–3.87; *P* = 0.011) for the risk of p53 mutation as compared with those with the APE1 Asp/Glu + Glu/Glu genotype after adjusting for covariates, including sex, smoking status, and tumor histology and stage (Table 3).

**Table 3.** Combined effects of hOGG1 and APE1 polymorphisms on p53 mutation in patients with lung cancer

Genes		No.	p53 mutation	OR (95% CI)	*P* ^a^
			No.		
hOGG1					
Ser/Ser		53	22	1	
Ser/Cys		106	50	1.33 (0.66–2.69)	0.430
Cys/Cys		58	25	1.20 (0.81–4.36)	0.664
Ser/Cys + Cys/Cys		164	75	1.26 (0.65–2.45)	0.496
APE1					
Glu/Glu		20	8	1	
Asp/Glu		105	39	0.97 (0.35–2.72)	0.953
Asp/Asp		92	50	1.99 (0.69–5.76)	0.200
Asp/Glu + Glu/Glu		125	47	1	
Asp/Asp		92	50	2.15 (1.19–3.87)	0.011
hOGG1 and APE1					
Ser/Ser	Asp/Glu + Glu/Glu	27	8	1	
Ser/Cys + Cys/Cys	Asp/Glu + Glu/Glu	98	39	2.08 (0.77–5.61)	0.148
Ser/Ser	Asp/Asp	26	14	3.29 (0.94–11.51)	0.062
Ser/Cys + Cys/Cys	Asp/Asp	66	36	3.72 (1.33–10.40)	0.012

^a^Adjusted for sex, smoking status, and tumor histology and stage.

We next examined the combined effects of hOGG1 and APE1 on p53 mutation in lung tumors after adjustment for covariates such as sex, smoking status, and tumor histology and stage. Participants with the Ser/Cys + Cys/Cys plus Asp/Asp had an OR of 3.72 (95% CI, 1.33–10.40; *P* = 0.012) for p53 mutation as compared with participants with the Ser/Ser plus Asp/Glu + Glu/Glu (Table 3). The combined effects of polymorphisms in both genes on p53 mutation were not evident in other combinations, including Ser/Cys + Cys/Cys plus Asp/Glu + Glu/Glu and Ser/Ser plus Asp/Asp (Table 3). Although differences in participants with the Ser/Ser plus Asp/Asp were not statistically significant, the OR for p53 mutation was 3.29 (95% CI, 0.94–11.51; *P* = 0.062; Table 3). The lack of statistical significance could have been the result of the lower number of participants in this subgroup.

### Effects of hOGG1 and APE1 polymorphisms on p53 mutation according to smoking status and tumor histology

Participants were stratified by sex, smoking status, and tumor histology to determine whether hOGG1 and APE1 polymorphisms had differing effects on p53 mutation. The hOGG1 polymorphism was not associated with p53 mutation in the 6 subgroups (Table 4). However, as shown in Table 5, the APE1 polymorphism was associated with p53 mutation in nonsmokers (OR, 3.45; 95% CI, 1.39–8.53; *P* = 0.007) and in squamous cell carcinoma samples (3.21; 1.38–7.47, *P* = 0.007). The association of APE1 polymorphism with the risk of p53 mutation was marginal in both women and men (Table 5). The OR for p53 mutation associated with APE1 polymorphism combined with hOGG1 polymorphism markedly increased, from 3.45 to 6.78 and from 3.21 to 8.11, in nonsmokers and squamous cell carcinoma, respectively (Table 6). However, combined effects of hOGG1 and APE1 polymorphisms on the risk of p53 mutation were not seen in participants with adenocarcinoma (Table 6).

**Table 4.** Effect of hOGG1 polymorphism on p53 mutation in patients with lung cancer, according to sex, smoking status, and tumor histology

Genes	No.	p53 mutation	OR (95% CI)	*P*
		No.		
Women				
Ser/Ser	14	3	1	0.655^a^
Ser/Cys + Cys/Cys	51	16	1.39 (0.33–5.96)	
Men				
Ser/Ser	39	19	1	0.550^a^
Ser/Cys + Cys/Cys	113	59	1.26 (0.59–2.70)	
Nonsmokers				
Ser/Ser	26	9	1	0.954^b^
Ser/Cys + Cys/Cys	78	27	1.03 (0.39–2.72)	
Smokers				
Ser/Ser	27	13	1	0.364^b^
Ser/Cys + Cys/Cys	86	48	1.52 (0.62–3.76)	
Adenocarcinoma				
Ser/Ser	28	11	1	0.562^c^
Ser/Cys + Cys/Cys	83	26	1.41 (0.44–4.50)	
Squamous cell carcinoma				
Ser/Ser	25	11	1	0.117^c^
Ser/Cys + Cys/Cys	81	49	2.10 (0.38–5.30)	

^a^Adjusted for smoking status and tumor histology and stage.

^b^Adjusted for sex and tumor histology and stage.

^c^Adjusted for sex, smoking status, and tumor stage.

**Table 5.** Effect of APE1 polymorphism on p53 mutation in patients with lung cancer, according to sex, smoking status, and tumor histology

Gene	No.	p53 mutation	OR (95% CI)	*P*
		No.		
Women				
Asp/Glu + Glu/Glu	44	10	1	0.056^a^
Asp/Asp	21	9	3.18 (0.97–10.43)	
Men				
Asp/Glu + Glu/Glu	81	37	1	0.069^a^
Asp/Asp	71	41	1.88 (0.95–3.70)	
Nonsmokers				
Asp/Glu + Glu/Glu	64	16	1	0.007^b^
Asp/Asp	40	20	3.45 (1.39–8.53)	
Smokers				
Asp/Glu + Glu/Glu	61	31	1	0.348^b^
Asp/Asp	52	30	1.45 (0.67–3.18)	
Adenocarcinoma				
Asp/Glu + Glu/Glu	63	19	1	0.337^c^
Asp/Asp	48	18	1.52 (0.65–3.59)	
Squamous cell carcinoma				
Asp/Glu + Glu/Glu	62	28	1	0.007^c^
Asp/Asp	44	32	3.21 (1.38–7.47)	

^a^Adjusted for smoking status and tumor histology and stage.

^b^Adjusted for sex and tumor histology and stage.

^c^Adjusted for sex, smoking status, and tumor stage.

**Table 6.** Combined effects of hOGG1 and APE1 polymorphisms on p53 mutation in nonsmokers and participants with squamous cell carcinoma and adenocarcinoma

Genes		No.	p53 mutation	OR (95% CI)	*P*
hOGG1	APE1		No.		
Nonsmokers					
Ser/Ser	Asp/Glu + Glu/Glu	11	2	1	
Ser/Cys + Cys/Cys	Asp/Glu + Glu/Glu	53	14	1.66 (0.31–8.83)	0.553^a^
Ser/Ser	Asp/Asp	15	7	3.39 (0.48–23.57)	0.217^a^
Ser/Cys + Cys/Cys	Asp/Asp	25	13	6.78 (1.03–44.56)	0.046^a^
Squamous cell carcinoma					
Ser/Ser	Asp/Glu + Glu/Glu	15	4	1	
Ser/Cys + Cys/Cys	Asp/Glu + Glu/Glu	47	24	4.11 (1.01–16.82)	0.049^b^
Ser/Ser	Asp/Asp	10	7	7.08 (1.04–48.21)	0.045^b^
Ser/Cys + Cys/Cys	Asp/Asp	34	25	8.11 (1.99–32.96)	0.003^b^
Adenocarcinoma					
Ser/Ser	Asp/Glu + Glu/Glu	12	4	1	
Ser/Cys + Cys/Cys	Asp/Glu + Glu/Glu	51	15	0.98 (0.24–4.07)	0.980^c^
Ser/Ser	Asp/Asp	16	7	1.70 (0.30–9.57)	0.544^c^
Ser/Cys + Cys/Cys	Asp/Asp	32	11	1.25 (0.26–5.45)	0.768^c^

^a^Adjusted for sex and tumor histology and stage.

^b^Adjusted for sex, smoking status, and tumor stage.

^c^Adjusted for sex, smoking status, and tumor stage.

## DISCUSSION

In this study, the prevalence of hOGG1 Cys/Cys was 26.7% (58 of 217) in Taiwanese with lung cancer. The distribution of hOGG1 Cys/Cys in this population was lower than in 2 previous studies of Taiwanese with lung cancer (39.8% and 36.9%). The prevalence of hOGG1 Cys/Cys in Japanese and Chinese adults with lung cancer ranged from 17.0% to 27.4% and from 16.9% to 36.1%, respectively. Interestingly, the prevalence of hOGG1 Cys/Cys in whites with lung cancer ranged from 2.7% to 18.2%, which is significantly lower than in Taiwanese, Japanese, and Chinese patient populations. The prevalence of the hOGG1 Cys/Cys genotype in this population was not higher than in other studies of Asian populations (eTable 2).

Substantial evidence indicates that hOGG1 Ser326Cys is associated with an increased risk of lung cancer.^16^^–^^20^ An early *in vitro* study provided cellular evidence that, as compared with the hOGG1 Ser/Ser genotype, the hOGG1-Cys variant results in less repair of 8-hydroxyguanine in damaged DNA.^29^ A more recent study confirmed this finding and also noted that the hOGG1-Cys variant resulted in less DNA glycosylase activity as compared with the hOGG1-Ser/Ser genotype. However, a biological function of hOGG1 Ser326Cys polymorphism was not supported, as the hOGG1-Cys variant was not associated with p53 mutation in patients with lung cancer.^30^ This discrepancy may have been due to the predominant influence of APE1 on the DNA glycosylase activity of hOGG1. In the present study, we consistently found that the hOGG1 Ser326Cys polymorphism was not associated with p53 mutation in the present population; however, p53 mutation was significantly more frequent in participants who had hOGG1-Cys alleles plus the APE1 Asp/Asp genotype. This finding lends support to the hypothesis that hOGG1 activity in the repair of oxidative damage to DNA is strongly regulated by APE1.

Findings regarding an association between the APE1 Asp148Glu polymorphism and lung cancer risk have been inconsistent. A recent study showed that lung cancer risk was higher for the APE1-Glu variant than for the APE1 Asp/Asp genotype,^24^ whereas other studies found no association between APE1 polymorphism and lung cancer risk.^16^^,^^21^^–^^23^ Another report found that lung cancer was lower for the APE1-Glu variant than for the APE1 Asp/Asp genotype, especially in smokers.^31^ In addition, individuals with advanced NSCLC who had the APE1-Glu variant had poorer responses to chemotherapy and radiotherapy than did those with the APE1 Asp/Asp genotype.^32^ An *in vitro* biochemical study found that endonuclease activity and DNA binding activity were unaffected by APE1 Asp148Glu polymorphism.^15^

In the present study, APE1 polymorphism was associated with p53 mutation in patients with squamous cell carcinoma, which is more common among smokers. However, an association between APE1 polymorphism and p53 mutation was seen only in nonsmokers (Table 5). Among nonsmokers, 8 of 10 Asp/Asp (80%) carriers versus 5 of 16 Asp/Glu + Glu/Glu (31%) carriers with p53 mutation had squamous cell carcinoma; however, 12 of 30 Asp/Asp carriers (40%) versus 11 of 48 Asp/Glu + Glu/Glu (23%) with p53 mutation had adenocarcinoma. Therefore, the association of APE1 polymorphism with p53 mutation in nonsmokers was higher among those with squamous cell carcinoma than in those with adenocarcinoma.

Combined APE1 and hOGG1 polymorphism markedly increased the risk of p53 mutation in nonsmokers and patients with squamous cell carcinoma (Table 6). However, combined polymorphism did not increase the risk of p53 mutation in patients with adenocarcinoma (Table 6). This could be due to the lower rate of p53 mutation in patients with adenocarcinoma as compared with squamous cell carcinoma patients (33% vs 57%, *P* = 0.001; Table 2). These results suggest that the effects of hOGG1 and APE1 polymorphism on the risk of p53 mutation differ according to smoking status and tumor histology. However, these findings should be verified in a larger study population.

In summary, the risk of p53 mutation was higher among Taiwanese lung cancer patients with the APE1 Asp/Asp genotype than in those with the APE1-Glu variant. In addition, participants with the APE1 Asp/Asp genotype combined with hOGG1-Cys variant had the highest risk of p53 mutation.

## ONLINE ONLY MATERIALS

eTables are available on the journal’s website at http://dx.doi.org/10.2188/jea.JE20120048.

eTables.
