# Supplementary material for: The APE1 Asp/Asp Genotype and the Combination of APE1 Asp/Asp and hOGG1-Cys Variants Are Associated With Increased p53 Mutation in Non–Small Cell Lung Cancer
Source: J Epidemiol. 2012 Nov 5;22(6):537–42. doi: 10.2188/jea.JE20120048 (PMC3798566; doi:10.2188/jea.JE20120048)
Supplement: eTables. [file je-22-537-s001.pdf]

eTable 1. Observed and expected genotypic frequencies of hOGG1 and APE1 polymorphisms in control group (n=206).

Gene	Observed n (%)	Expected n (%)	p (HWE)
hOGG1			
Ser/Ser	76 (37)	74.6 (36)	0.69
Ser/Cys	96 (47)	98.7 (48)	
Cys/Cys	34 (16)	32.6 (16)	
APE1			
Asp/Asp	78 (39)	74.6 (37)	0.33
Asp/Glu	92 (43)	98.7 (48)	
Glu/Glu	36 (18)	32.6 (15)	

HWE, Hardy-Weinberg equilibrium

eTable 2. Prevalence of hOGG1 Ser326Cys genotypes in the present participants and in studies of Asian and white populations.

Ref. No.	Population	No. (%)			
		Ser/Ser	Ser/Cys	Cys/Cys	Ser/Cys + Cys/Cys
1	Japanese	16 (35.6)	19 (42.2)	10 (22.2)	29 (64.4)
2	Japanese	85 (35.3)	115 (47.7)	41 (17.0)	156 (64.7)
3	Japanese	40 (29.0)	71 (51.4)	27 (19.6)	98 (71.0)
4	Japanese	54 (27.3)	106 (53.5)	38 (19.2)	144 (72.7)
5	Japanese	285 (26.0)	544 (49.6)	268 (24.4)	812 (74.0)
6	Japanese	117 (22.7)	257 (49.9)	141 (27.4)	398 (77.3)
7	Japanese	27 (25.0)	55 (50.9)	26 (24.1)	81 (75.0)
8	Korean	40 (20.0)			160 (80.0)
9	Korean	46 (27.7)	67(40.4)	53(31.9)	120 (72.3)
10	Chinese	37 (31.4)	61 (51.7)	20 (16.9)	81 (68.6)
11	Chinese	27 (11.9)	132 (58.1)	68 (30.0)	200 (88.1)
12	Chinese	83 (18.2)	208 (45.7)	164 (36.1)	372 (81.8)
13	Chinese	100 (17.2)	288 (49.6)	193 (33.2)	481 (82.8)
14	Taiwanese	142 (13.0)	518 (47.2)	436 (39.8)	954 (87.0)
15	Taiwanese	68 (19.0)	158 (44.1)	132 (36.9)	290 (81.0)
16	Whites	68 (64.8)	32 (30.5)	5 (4.8)	37 (35.2)
17	Whites	149(58.2)	93 (36.3)	14 (5.5)	107 (41.8)
18	Whites	1401 (65.0)	661 (30.7)	93 (4.3)	754 (35.0)
19	Whites	144 (57.4)	93 (37.0)	14 (5.6)	107 (42.6)
20	Whites	254 (58.9)	155 (36.0)	22 (5.1)	177 (41.1)
21	Whites	182 (55.8)	100 (30.7)	44 (13.5)	144 (44.2)
22	Whites	74 (67.3)	33 (30.0)	3 (2.7)	36 (32.7)
23	Whites	48 (54.5)	24 (27.3)	16 (18.2)	40 (45.5)
24	Whites	101 (56.4)	65 (36.3)	13 (7.3)	78 (43.6)
25	Whites	90 (63.8)	45 (31.9)	6 (4.3)	51 (36.2)
Present study	Taiwanese	53 (24.5)	106 (48.8)	58 (26.7)	164 (75.5)

eTable 2 references

1. Kohno T, Shinmura K, Tosaka M, Tani M, Kim SR, Sugimura H, et al. Genetic polymorphisms and alternative splicing of the hOGG1 gene, that is involved in the repair of 8-hydroxyguanine in damaged DNA. Oncogene. 1998;16:3219-25.
2. Sugimura H, Kohno T, Wakai K, Nagura K, Genka K, Igarashi H, et al. hOGG1 Ser326Cys polymorphism and lung cancer susceptibility. Cancer Epidemiol Biomarkers Prev. 1999;8:669-74.
3. Ito H, Hamajima N, Takezaki T, Matsuo K, Tajima K, Hatooka S, et al. A limited association of OGG1 Ser326Cys polymorphism for adenocarcinoma of the lung. J Epidemiol. 2002;12:258-65.
4. Sunaga N, Kohno T, Yanagitani N, Sugimura H, Kunitoh H, Tamura T, et al. Contribution of the NQO1 and GSTT1 polymorphisms to lung adenocarcinoma susceptibility. Cancer Epidemiol Biomarkers Prev. 2002;11:730-8.
5. Kohno T, Kunitoh H, Toyama K, Yamamoto S, Kuchiba A, Saito D, et al. Association of the OGG1-Ser326Cys polymorphism with lung adenocarcinoma risk. Cancer Sci. 2006;97:724-8.
6. Okasaka T, Matsuo K, Suzuki T, Ito H, Hosono S, Kawase T, et al. hOGG1 Ser326Cys polymorphism and risk of lung cancer by histological type. J Hum Genet. 2009;54:739-45.
7. Miyaishi A, Osawa K, Osawa Y, Inoue N, Yoshida K, Kasahara M, et al. MUTYH Gln324His gene polymorphism and genetic susceptibility for lung cancer in a Japanese population. J Exp Clin Cancer Res. 2009;28:10-6.
8. Lee CH, Lee KY, Choe KH, Hong YC, Noh SI, Eom SY, et al. [Effects of oxidative DNA damage and genetic polymorphism of the glutathione peroxidase 1 (GPX1) and 8-oxoguanine glycosylase 1 (hOGG1) on lung cancer]. J Prev Med Public Health. 2006;39:130-4.
9. Cho S, Kim MJ, Choi YY, Yoo SS, Lee WK, Lee EJ, et al. Associations between polymorphisms in DNA repair genes and TP53 mutations in non-small cell lung cancer. Lung Cancer. 2011;73:25-31.
10. Lan Q, Mumford JL, Shen M, Demarini DM, Bonner MR, He X, et al. Oxidative damage-related genes AKR1C3 and OGG1 modulate risks for lung cancer due to exposure to PAH-rich coal combustion emissions. Carcinogenesis. 2004;25:2177-81.
11. Liang G, Pu Y, Yin L. Rapid detection of single nucleotide polymorphisms related with lung cancer susceptibility of Chinese population. Cancer Lett. 2005;223:265-74.
12. Li Z, Guan W, Li MX, Zhong ZY, Qian CY, Yang XQ, et al. Genetic polymorphism of DNA base-excision repair genes (APE1, OGG1 and XRCC1)

and their correlation with risk of lung cancer in a Chinese population. Arch Med Res. 2011;42:226-34.

13. Qian B, Zhang H, Zhang L, Zhou X, Yu H, Chen K. Association of genetic polymorphisms in DNA repair pathway genes with non-small cell lung cancer risk. Lung Cancer. 2011;73:138-46.
14. Chang CH, Hsiao CF, Chang GC, Tsai YH, Chen YM, Huang MS, et al. Interactive effect of cigarette smoking with human 8-oxoguanine DNA N-glycosylase 1 (hOGG1) polymorphisms on the risk of lung cancer: a case-control study in Taiwan. Am J Epidemiol. 2009;170:695-702.
15. Liu CJ, Hsia TC, Tsai RY, Sun SS, Wang CH, Lin CC, et al. The joint effect of hOGG1 single nucleotide polymorphism and smoking habit on lung cancer in Taiwan. Anticancer Res. 2010;30:4141-5.
16. Wikman H, Risch A, Klimek F, Schmezer P, Spiegelhalder B, Dienemann H, et al. hOGG1 polymorphism and loss of heterozygosity (LOH): significance for lung cancer susceptibility in a caucasian population. Int J Cancer. 2000;88:932-7.
17. Vogel U, Nexo BA, Wallin H, Overvad K, Tjonneland A, Raaschou-Nielsen O. No association between base excision repair gene polymorphisms and risk of lung cancer. Biochem Genet. 2004;42:453-60.
18. Hung RJ, Brennan P, Canzian F, Szeszenia-Dabrowska N, Zaridze D, Lissowska J, et al. Large-scale investigation of base excision repair genetic polymorphisms and lung cancer risk in a multicenter study. J Natl Cancer Inst. 2005;97:567-76.
19. Loft S, Svoboda P, Kasai H, Tjonneland A, Vogel U, Moller P, et al. Prospective study of 8-oxo-7,8-dihydro-2'-deoxyguanosine excretion and the risk of lung cancer. Carcinogenesis. 2006;27:1245-50.
20. Sorensen M, Raaschou-Nielsen O, Hansen RD, Tjonneland A, Overvad K, Vogel U. Interactions between the OGG1 Ser326Cys polymorphism and intake of fruit and vegetables in relation to lung cancer. Free Radic Res. 2006;40:885-91.
21. Zienolddiny S, Campa D, Lind H, Ryberg D, Skaug V, Stangeland L, et al. Polymorphisms of DNA repair genes and risk of non-small cell lung cancer. Carcinogenesis. 2006;27:560-7.
22. De Ruyck K, Szaumkessel M, De Rudder I, Dehoorne A, Vral A, Claes K, et al. Polymorphisms in base-excision repair and nucleotide-excision repair genes in relation to lung cancer risk. Mutat Res. 2007;631:101-10.
23. Janik J, Swoboda M, Janowska B, Ciesla JM, Gackowski D, Kowalewski J, et al. 8-Oxoguanine incision activity is impaired in lung tissues of NSCLC patients with the polymorphism of OGG1 and XRCC1 genes. Mutat Res. 2011;709-710:21-31.
24. Park J, Chen L, Tockman MS, Elahi A, Lazarus P. The human 8-oxoguanine DNA

N-glycosylase 1 (hOGG1) DNA repair enzyme and its association with lung cancer risk. Pharmacogenetics. 2004;14:103-9.

25. Hu YC, Ahrendt SA. hOGG1 Ser326Cys polymorphism and G:C-to-T:A mutations: no evidence for a role in tobacco-related non small cell lung cancer. Int J Cancer. 2005;114:387-93.
